# Mapping BOLD Activation by Pharmacologically Evoked Tremor in Swine

**DOI:** 10.3389/fnins.2019.00985

**Published:** 2019-09-18

**Authors:** Jeyeon Lee, Hang Joon Jo, Inyong Kim, Jihyun Lee, Hoon-Ki Min, Myung-Ho In, Emily J. Knight, Su-Youne Chang

**Affiliations:** ^1^Department of Neurologic Surgery, Mayo Clinic, Rochester, MN, United States; ^2^Department of Radiology, Mayo Clinic, Rochester, MN, United States; ^3^Department of Neurology, Mayo Clinic, Rochester, MN, United States; ^4^Department of Physiology, College of Medicine, Hanyang University, Seoul, South Korea; ^5^Laboratory of Brain and Cognitive Sciences for Convergence Medicine, Hallym University College of Medicine, Anyang, South Korea; ^6^Department of Developmental Behavioral Pediatrics, University of Rochester, Rochester, NY, United States; ^7^Department of Physiology and Biomedical Engineering, Mayo Clinic, Rochester, MN, United States

**Keywords:** tremor, essential tremor, harmaline-induced tremor, pharmacological fMRI, swine model, pig

## Abstract

Harmaline-induced tremor is one of the most commonly utilized disease models for essential tremor (ET). However, the underlying neural networks involved in harmaline-induced tremor and the degree to which these are a representative model of the pathophysiologic mechanism of ET are incompletely understood. In this study, we evaluated the functional brain network effects induced by systemic injection of harmaline using pharmacological functional magnetic resonance imaging (ph-fMRI) in the swine model. With harmaline administration, we observed significant activation changes in cerebellum, thalamus, and inferior olivary nucleus (ION). In addition, inter-regional correlations in activity between cerebellum and deep cerebellar nuclei and between cerebellum and thalamus were significantly enhanced. These harmaline-induced effects gradually decreased with repeated administration of drug, replicating the previously demonstrated ‘tolerance’ effect. This study demonstrates that harmaline-induced tremor is associated with activity changes in brain regions previously implicated in humans with ET. Thus, harmaline-induction of tremor in the swine may be a useful model to explore the neurological effects of novel therapeutic agents and/or neuromodulation techniques for ET.

## Introduction

Essential tremor (ET) is the most prevalent form of pathologic tremor and one of the most common adult-onset neurologic impairments (Louis and Ferreira, 2010). The incidence of ET in individuals age 60 and older is estimated to be as high as 6.3–9% (Pahwa and Lyons, 2003) and it affects as many as 10 million people in the United States (Louis and Ferreira, 2010). Despite ET’s high prevalence and disabling effects, available pharmacological interventions are only helpful for about 50% of patients with ET, and nearly one out of three patients stop taking their medications due to adverse effects. The development of new therapies has been hampered by a lack of knowledge about tremor pathophysiology and the lack of a fully validated preclinical model for evaluating potential tremor-suppressing drugs.

One animal model that has been proposed in the literature for preclinical screening of novel ET therapies is the induction of tremor using harmaline, a beta-carboline derivative. The utility of this model is largely related to the fact that the harmaline-induced tremor shares several characteristics with ET (Wilms et al., 1999; Martin et al., 2005; Handforth, 2012). First, the peak frequency of harmaline-induced tremor in animals is 8–16 Hz, which is similar to the frequency of ET in humans (Handforth, 2012). Additionally, similar to the predominance of action-based tremor in ET, harmaline-induced tremor is particularly visible when the animal ambulates and is attenuated when the animal is at rest (Handforth, 2012). Perhaps most critically, the harmaline-induced tremor is reduced by medications that also show clinical efficacy in treatment of patients with ET (Martin et al., 2005; Paterson et al., 2009).

However, there remains significant controversy over the degree to which harmaline induction of tremor recapitulates the actual underlying pathophysiology of ET. In support of harmaline as a valid preclinical model of the pathophysiology of ET, serum harmane, another type of beta-carboline derivative, is elevated in ET patients correlated with cerebellar neuronal dysfunction or degeneration (Louis et al., 2007). In addition, considerable overlap has been demonstrated in neural networks implicated in both harmaline-induced tremor and ET. For one the olivocerebellar system comprised of inferior olivary nucleus (ION), cerebellar cortex, and deep cerebellar nuclei (DCN) is thought to play a key role in tremor generation in both types of tremor. In patients with ET, studies using positron emission tomography (PET), functional MRI and MR-spectroscopy all describe a relationship between olivocerebellar pathway and tremor (Hallett and Dubinsky, 1993; Wills et al., 1994; Boecker et al., 1996; Bucher et al., 1997; Louis et al., 2002). Likewise, microinjections of harmaline into the ION have the ability to generate sustained rhythmic neural activity in this area (Montigny and Lamarre, 1975) which is likely the origin of harmaline-induced tremor. It has also been shown that tremor responses to harmaline administration can be eliminated by either destruction of ION by systemic 3-acetylpyridine injection (Simantov et al., 1976) or selective knock-down of the CaV3.1 gene in the ION (Park et al., 2010), suggesting that the ION is critical to tremor induction by harmaline. Furthermore, alcohol administration, which is known to improve tremor in patients with ET, suppresses cerebellar hypermetabolism and increases ION blood flow to a greater extent in patients with ET versus controls. Finally, the ability to acquire conditioned eye blink responses, which is known to depend on the olivocerebellar pathway, is significantly reduced in ET patients compared with normal controls (Martin et al., 2005). Overall, the similarity in involvement of the ION and olivocerebellar network in both harmaline-induced tremor and ET constitute mounting support for harmaline as a valid preclinical model for ET.

However, there remain some unresolved discrepancies between ET and harmaline-induced tremor which necessitate further investigation. These are largely related to controversy surrounding the degree of thalamic involvement in the two types of tremor. In ET patients, it has been reported that the cerebello-thalamo-cortical pathways play a crucial role (Bucher et al., 1997; Buijink et al., 2015b; Gallea et al., 2015). This is supported by the fact that either thalamotomy or thalamic deep brain stimulation (DBS) dramatically suppresses ET (Flora et al., 2010). A number of functional imaging studies also point to thalamic involvement during tremor (Jenkins et al., 1993; Wills et al., 1994; Bucher et al., 1997). In contrast, it is speculated that the brainstem and spinal cord are sufficient to express tremor in the harmaline model. Previous report has shown that cooling of the motor cerebral cortex and lesions of the ventrolateral thalamus or globus pallidus do not affect harmaline induced-tremor in normal monkeys (Lamarre et al., 1975). Moreover, an intercollicular decerebration does not diminish the harmaline-induced tremor in cats or monkeys (Battista et al., 1970; Villablanca and Riobó, 1970). These findings imply that harmaline-induced tremor-related activity in the olivocerebellar system can be directly transmitted to the spinal cord and mediated via cerebellar nuclei and the lower brainstem without the involvement of thalamus and motor cortex. However, this evidence remains inconclusive as a recent study showed that thalamic DBS can still be effective at suppressing harmaline-induced tremor in the rodent (Bekar et al., 2008).

This study aims to clarify thalamic involvement in harmaline-induced tremor through application of two innovative technologies. We employ pharmacological fMRI (ph-MRI) techniques in a swine model of harmaline-induced tremor (Lee et al., 2018). The swine model is useful because it shares greater homology with the human brain in both thalamic composition and global connectivity than the rodent model and is significantly less expensive than the non-human primate, thus enabling larger sample sizes while producing results that are easily translatable to human patients. ph-fMRI has emerged as an alternative or complementary means of assessing the neural mechanisms of drug action since it can investigate effects of pharmacological agents at a network level and remotely from regions of highest target receptor densities (Bruinsma et al., 2018). Hence ph-fMRI enables a “system evaluation” of underlying effects of a drug on brain networks, independent of its biochemical mechanism of action (Wandschneider and Koepp, 2016). Here, we unite these two recent technological advances to elucidate cerebral BOLD activation induced by harmaline and to define changes in functional connectivity among specific regions of interest (ROI) within the olivo-cerebello-thalamo-cortical motor circuitry.

## Materials and Methods

### Animals

All study procedures were performed in accordance with the Guide for the Care and Use of Laboratory Animals in an AAALAC, International-accredited facility and approved by the Mayo Clinic Institutional Animal Care and Use Committee. The subject groups consisted of five normal (*n* = 5), domestic male swine weighing 35 ± 5 Kg. Animals were socially housed in pairs and animal enclosures were cleaned daily and sanitized weekly before feeding. The animals were fed standard chow (Purina Animal Nutrition, LLC, Shoreview, MN) as recommended by the attending veterinarian and provided *ad libitum* access to tap water. Enrichment was provided in the form of twice weekly rotation of toys and daily treats. Records of cleaning schedules as well as temperature and humidity recordings were monitored daily. Each animal was checked daily by the animal care staff and 3 days per week by the Mayo Clinic veterinarians.

### Experimental Procedure of Alternative fMRI and Tremor Monitoring Within Subjects

**FIGURE 1:**
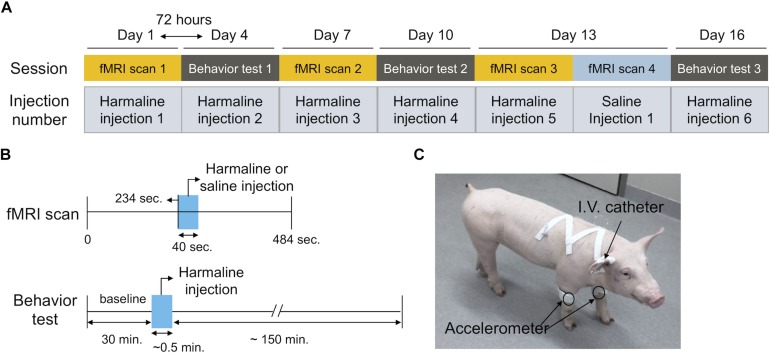
Experimental timeline. **(A)** *Experimental design*. The experiment consisted of six sessions in total comprised of three fMRI scans and three interleaved behavioral tests. The interval between sessions was three days (72 h). Harmaline injection was performed at every session (total six injections per animal) Saline injection was performed during the final fMRI session immediately following the final harmaline injection fMRI scan **(B)** *Session timeline*. The duration of the fMRI scan was 484 s. Harmali ne was administered intravenously and it was initiated at *t* = 234 s, with a duration of 40 s. Behavioral testing sessions (total duration 3 h each) included a 30 min baseline, followed by harmaline injection, and 2 h post-injection monitoring period. **(C)** *Behavioral setup.* Accelerometers were attached over bilateral front legs to monitor tremor activity. Drug delivery was performed intravenously through a venous indwelling catheter.

The experiment consisted of seven sessions in total, including four fMRI scans (three with harmaline injection and one saline control) and three behavioral tests. The fMRI scans and behavior sessions were performed alternately and the interval between sessions was 72 h (Figure 1A). Harmaline injection was performed in three fMRI and three tremor monitoring sessions, equivalent to six harmaline injections per animal. The saline injection control was performed within the same day and immediately following the final harmaline fMRI scan.

For fMRI sessions, the animal was sedated with Telazol (5 mg/kg) and Xylazine (2 mg/kg) intramuscularly and a venous indwelling catheter was inserted into an ear vein for harmaline administration. Anesthesia was continuously maintained with 1.5–2% isoflurane and the vital signs (heart rate: ∼120 bpm, respiration: 12 breaths per minute and temperature 36–37°C) were monitored during scanning. An in-house developed and fabricated MR-compatible stereotactic head frame designed for large animals was used for scanning. Harmaline (harmaline hydrochloride dihydrate, Sigma-Aldrich, St. Louis, MO) was administered intravenously through the ear vein at a dose of 6 mg/kg dissolved in sterile saline (10 ml). The solution was freshly prepared immediately prior to the injection (Lee et al., 2018).

For the tremor monitoring, we followed the experimental procedures previously described (Lee et al., 2018). Briefly, an animal was temporary sedated with ketamine (10 mg/Kg) and medetomidine (0.2 mg/Kg) intramuscularly and a venous indwelling catheter was inserted. The accelerometer was attached with an elastic vet bandage, while the animal was under sedation. Once all equipment was attached, the pig was awakened by atipamezole administration (1 mg/Kg, i.v.). Once the animal was able to ambulate and maintain balance, baseline motion was monitored with the attached accelerometer for 30 min. Then, harmaline was administered intravenously at a dose of 6 mg/kg dissolved in sterile saline (10 ml). Accelerometer monitoring was performed for a maximum of 3 h (Figures 1B,C).

### MRI Acquisition

Anatomical and fMRI images were acquired in all animals on a 3T Signa Excite MRI scanner (16.0M4, GE Medical Systems, Milwaukee, WI) using a homemade 6-channel receive-only phased-array surface coil composed of linear arrays of loop coils with a dimension of 23 × 8 cm. Anatomical 3D MPRAGE volume was acquired with the following imaging protocols: TI/TR/TE = 1000/8.06/3.30 ms, flip angle = 8°, slice thickness = 0.8 mm, matrix size = 300 × 300 × 108, FOV = 240 × 240 × 87 mm^3^ and average = 2. The imaging parameters for gradient-echo EPI data were: TR/TE = 2000/40 ms, no partial echo, parallel imaging acceleration factor = 2, 19 slices, voxel resolution = 1.7 × 1.7 × 2.4 mm^3^. The scan times for MPRAGE and fMRI were 25 min 36 s and 8 min 4 s, respectively.

### Preprocessing of fMRI Data

**FIGURE 2:**
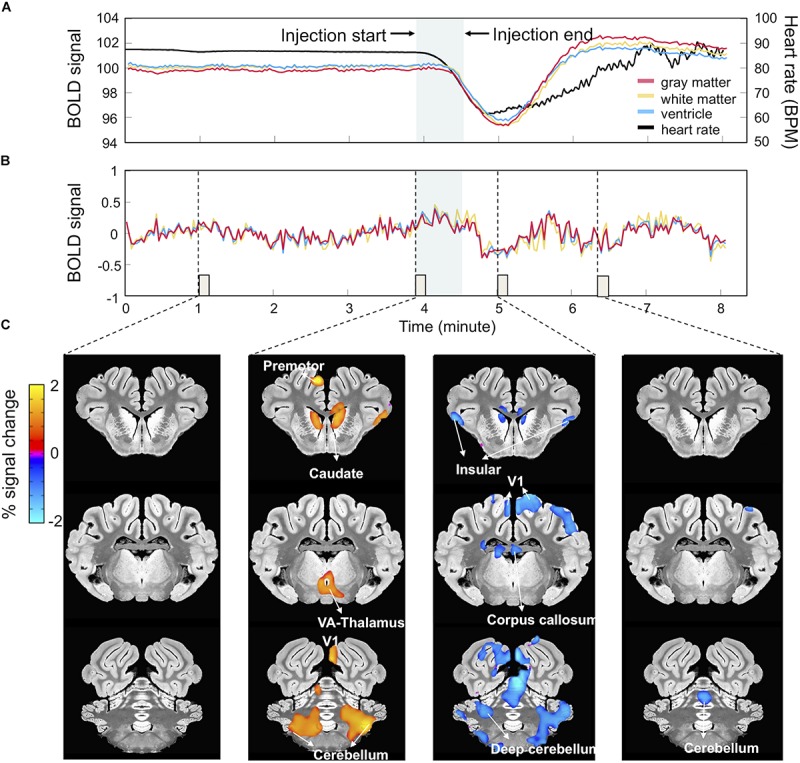
Harmaline-evoked physiological changes and associated BOLD signal fluctuations. **(A)** *Harmaline-induced heart rate change*. Harmaline injection (*t* = 234 s, duration = 40 s) induced a significant decrease in heart rate (black) with subsequent slow recovery to pre-injection baseline. Associated with this relative bradycardia were large drifts in the mean BOLD signal derived from gray (red), white (yellow), and ventricular matter (blue). **(B)** *BOLD signal traces after artifact removal.* A regression analysis was performed using heart rate waveform as regressor, resulting in a relatively stable BOLD signal trace. **(C)** *BOLD signal activation maps after artifact removal.* Percent signal changes of BOLD signal at specific time points (each column matched with an 8-s epoch indicated by dotted lines and small boxes in **B**) throughout the session reveal regional BOLD signal changes related to the time course of harmaline injection.

Preprocessing of all imaging data was conducted using the Analysis of Functional NeuroImages (AFNI) software package (Cox, 1996; Cox and Jesmanowicz, 1999). This includes despiking, slice-timing and motion correction, spatial smoothing with a gaussian kernel (full width at half maximum = 2.8 mm), and co-registration to the high-resolution pig brain atlas (Saikali et al., 2010) with the cost function of the Hellinger metric (Jo et al., 2009). After co-registration of the mean EPI for the time series to the MPRAGE, the MPRAGE was co-registered to the pig brain atlas. The combined transformation matrix was applied to all of the EPI series. To eliminate the cardiovascular artifact induced by drug injection (Figure 2), we used the individual heart rate as a nuisance regressor. Functional activation maps were generated for each subject by general linear model (GLM) regression between the observed BOLD signal intensity changes in each voxel and a hemodynamic response function with a temporal block length of 25 s. For group level analysis, one sample *t*-test was used to produce a statistical map of harmaline-induced activation across the entire experiment (FDR corrected, *q* < 0.0005). To assess for development of tolerance to harmaline injection across sessions, an individual activation map for each session was generated by paired *t*-test comparing the BOLD signal changes induced by harmaline with those induced by saline control using an uncorrected threshold of *p* < 0.005. The signal percentage changes of the BOLD time series were also calculated for each individual.

### Functional Correlation Analysis in Regions of Interest Level

To examine whether harmaline injection can induce changes in functional network connectivity, inter-ROI correlation matrices were calculated based on averaged EPI time series per ROI which was derived from a pig brain atlas (Saikali et al., 2010). The ROI included sensorimotor cortex (SMC), thalamus, limbic area, cerebellum, deep cerebellar nuclei (DCN), and visual cortex. In order to verify the change in functional correlation among these areas due to harmaline injection, the timeline was divided into a pre-injection period (0∼232 s) and post-injection period (234∼484 s), and the difference of the inter-ROI correlation matrix between each time interval was calculated. Post-saline injection matrices were subtracted from post-harmaline injection matrices to isolate functional connectivity changes specific to harmaline injection. The functional correlation was computed as partial correlation coefficient to subtract mutual dependencies on common influences from other brain areas allowing access to a quantity more closely related to direct interaction (Marrelec et al., 2006). The statistical significance of the correlation for each region was validated by the permutation method. For example, to estimate a null distribution of the correlation value between two ROIs, the sample order of one ROI was randomly shuffled (*n* = 2,000), while the order of another ROI remained unchanged. Then, we estimated the statistical significance by comparing the correlation value obtained by the real signal and the null distribution derived from the permutation.

### Tremor Analysis

Tremor monitoring and analysis was performed according to the previously published method (Lee et al., 2018). Briefly, motion activity was measured with an in-house fabricated wireless movement sensor and the motion power was transmitted wirelessly (Figure 1C). Data were sampled at 100 Hz and Fast Fourier Transform (FFT) was performed by amplitude of accelerometer data in hanning windowed 10-s bins to obtain power spectra in the frequency domain. The averaged spectral power was calculated between 0 and 10 Hz (non-tremor motion spectrum) and between 10 and 16 Hz (the harmaline tremor frequency bandwidth), and a tremor index then was derived as the mean amplitude of tremor frequency (10–16 Hz) divided by mean value of non-tremor motion spectrum (0–10 Hz). For visualization, 10-s bin tremor indexes were then averaged across 10 min time windows and each index within these 10 min windows was normalized based on the mean value derived from the 30 min baseline.

## Results

### Physiological Artifact Induced by Harmaline Injection

Drugs can influence the BOLD signal both at a neuronal and vascular level complicating the interpretation of the effects observed (Wandschneider and Koepp, 2016). Cardiac responses induced by harmaline injection have been reported in previous studies (Aarons et al., 1977; Berrougui et al., 2006). Consistently, in this experiment, we observed that heart rate dramatically decreased immediately after harmaline injection, reached a minimum at around 10 min after harmaline injection, and then gradually recovered to the same level as before injection (Figure 2A). These changes in heart rate were associated with a large drift in the averaged traces of the BOLD signal in the gray matter, white matter and ventricle areas (Figure 2A). The BOLD signal decreased after the harmaline injection, reached a minimum, and then slowly recovered, thus showing a similar pattern to the cardiac response albeit slightly delayed (Figure 2A). The cardiac response and the induced artifact in the BOLD signal were observed during all three MRI sessions (Supplementary Figure S1).

As described in methods, to eliminate these physiological artifacts (Figure 2), we measured the individual heart rate throughout the entire scanning session and using the individual heart rate waveform as a regressor, computed a residual signal. Figure 2B illustrates the cleaned signal trace after regression which is stable without a cardiac artifact. Figure 2C depicts the activation maps obtained by setting the ideal reference function over 8 s blocks corresponding to the specific time points marked by the dotted lines in Figure 2B. As shown in Figure 2C, activation patterns related to harmaline injection appeared at each time point.

### Harmaline Induced Activation Map

**FIGURE 3:**
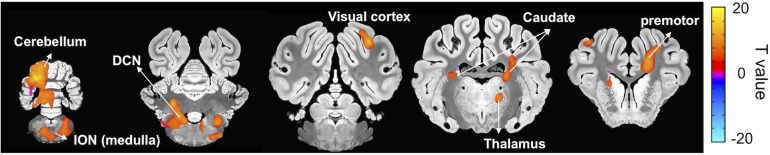
Brain areas exhibiting significant harmaline-induced activation across entire experiment. Areas demonstrating significant BOLD activation with harmaline injection include ION, cerebellum, DCN, specific thalamus regions, and premotor cortex as well as caudate and visual cortex (One-sample *t*-test, *q* < 0.0005, FDR corrected) (Table 1).

Once the BOLD responses were adjusted for physiological changes induced by harmaline (Figure 2), an overall harmaline-induced activation map across all sessions was generated. Since the ION has been previously speculated as the predominant brain region involved in harmaline-induced tremor (Montigny and Lamarre, 1975; Park et al., 2010), we defined the most effective block length for analysis as that which demonstrated maximal activation of the ION. As a result, we selected 25 s as the regression block length for primary analyses (Supplementary Figure S2). Importantly, although the magnitude of activation and number of vowels varied with block length, the brain regions activated were largely consistent across various block lengths. We observed significant activity in olivo-cerebello-thalamo-cortical loop, including ION (medulla), cerebellum, DCN and thalamus (*q* < 0.0005, one-sample *t*-test). Moreover, significant BOLD increase was also observed in premotor cortex, caudate, and visual cortex (Figure 3 and Table 1).

**TABLE 1:** Peak coordinates of activated clusters induced by harmaline injection across entire experiment.

**Hemisphere**	**Area**	***x***	***y***	***z***	**Cluster size**	***t*-value**
L/R	Cerebellum; Deep cerebellar nucleus	5.6	23.9	7.1	390	19.9
L/R	Olivary nucleus; medulla	–5.6	19.7	–12.4	160	14.38
R	Secondary visual cortex (V2)	–12.6	9.9	21.1	49	22.64
R	Ventral anterior thalamic nucleus; Caudate nucleus	–4.2	–9.7	1.5	189	12.57
R	Caudate nucleus; Premotor Cortex	–15.6	2.9	7.1	319	16.74
R	Prepiriform area	–12.6	–26.5	–1.2	28	11.73
L	Primary Somatosensory Cortex	15.4	–19.5	21.1	22	11.85

*L, left; R, right.*

**FIGURE 4:**
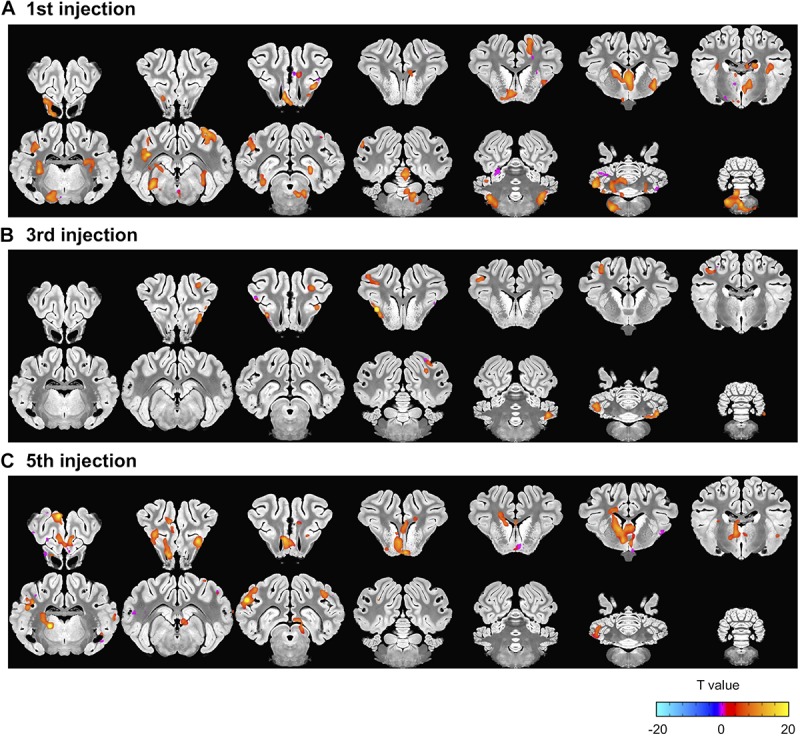
Decrement in harmaline-induced brain activity within individual sessions. **(A–C)** Brain areas exhibiting greater activation with harmaline as compared to saline control at 1st, 3rd and 5th injection, respectively (paired *t*-test, *p* < 0.005, uncorrected) (Tables 2A-C).

Next, we explored the change in harmaline-induced activation across subsequent sessions. We derived activation maps based on block design for each session (Figure 4). Notably, the activity in ION, cerebellum and DCN that was significantly enhanced in first injection (Figure 4A) gradually decreased or disappeared with repeated harmaline administration (Figures 4B,C and Tables 2A–C).

**TABLE 2A:** Peak coordinates of activated clusters induced by harmaline injection at first injection.

**Hemisphere**	**Area**	***x***	***y***	***z***	**Cluster size**	***t*-value**
L/R	Olivary nucleus; medulla; Cerebellum	4.2	21.1	–11.1	250	30.13
R	Cerebellum	–1.4	11.3	7.1	24	16.23
R	Cerebellum	–1.4	8.5	–5.4	90	22.9
L	Cerebellum	16.8	18.3	1.5	42	16.91
R	Cerebellum	–14	12.7	–8.2	56	17.03
L	Secondary visual cortex (V2)	19.6	11.3	18.3	23	11.79
R	Secondary visual cortex (V2)	–15.4	2.9	21.1	59	18.13
L	Geniculate nuclei; Hippocampus; Parahippocampal cortex	12.6	1.5	–1.2	69	18.37
R	Hippocampus	–9.8	4.3	7.1	36	13.43
L/R	Ventral anterior thalamic nucleus; Caudate nucleus	–1.4	–12.5	1.5	178	19.15
L	Mamillary body	1.4	5.5	–6.9	39	17.64
R	Primary Motor Cortex	–7	–18.1	22.5	34	10.69
R	Anterior prefrontal cortex	–7	–41.9	5.8	53	18.37
R	Amygdala	–11.2	–11.1	0.1	48	23.96
L	Somatosensory Association Cortex; Auditory Cortex	14	0.1	14.1	160	26.7
L	Prepiriform area	8.4	–39.1	–6.9	59	14.86

*L, left; R, right.*

**TABLE 2B:** Peak coordinates of activated clusters induced by harmaline injection at third injection.

**Hemisphere**	**Area**	***x***	***y***	***z***	**Cluster size**	***t*-value**
R	Cerebellum	-14	19.7	–2.7	50	14.61
L	Cerebellum	15.4	21.1	2.9	29	30.58
R	Primary visual cortex (V1)	–11.2	11.3	18.3	21	9.46
L	Somatosensory Association Cortex	16.8	–9.7	21.1	61	11.06
R	Primary Somatosensory Cortex	–9.8	–32.1	12.8	23	8.58
R	Prepiriform area	–9.8	–32.1	12.8	24	14.91
R	Insular cortex	–12.6	–29.3	1.5	20	8.29

*L, left; R, right.*

**TABLE 2C:** Peak coordinates of activated clusters induced by harmaline injection at fifth injection.

**Hemisphere**	**Area**	***x***	***y***	***z***	**Cluster size**	***t*-value**
L	Cerebellum	15.4	18.3	–1.2	30	41.74
R	Secondary visual cortex (V2)	–15.4	4.3	21.1	20	11.73
R	Parahippocampal cortex	–19.6	–5.5	–1.2	24	10.87
R	Anterior entorhinal cortex	–4.2	4.3	7.1	40	14.92
L	Insular cortex	11.2	–33.5	1.5	29	12.04
L	Auditory Cortex	16.8	–2.7	14.1	28	9.74
L	Caudate nucleus; Anterior prefrontal cortex; Reticular thalamic nucleus	4.2	–11.1	2.9	394	63.48
L	Dorsolateral prefrontal cortex; Dorsal anterior cingulate cortex	2.8	–36.3	11.4	120	40.28
L	Superior temporal gyrus	21	5.7	17	75	37.97
L	Ventral anterior thalamic nucleus	7	–4.1	4.4	41	29.61
R	Prepiriform area	–9.8	–32.1	–1.2	40	19.05

*L, left; R, right.*

### Correlation Between Harmaline-Induced Activation and Tremor

**FIGURE 5:**
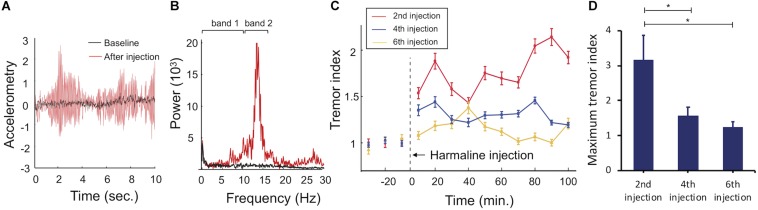
Accelerometer monitoring of harmaline-induced tremor. **(A)** *Raw accelerometer data.* Representative raw accelerometer tracing from a single animal, including baseline (black) and tremor response to harmaline injection (red). **(B)** *Fourier transformed motion data.* Tremor frequency was between 10 and 16 Hz (band 2, red) with a prominent peak at 13 Hz. Baseline motion (band 1, black) was between 0 and 10 Hz. **(C)** *Tremor index versus time*. Tremor index was computed as the mean amplitude of tremor frequency (10–16 Hz) divided by mean value of non-tremor frequency range (0–10 Hz). Tremor indices were calculated for each 10 s bin and averaged across 10 min epochs for visualization across each behavioral session (2nd harmaline injection = red line, 4th harmaline injection = blue line, 6th harmaline injection = yellow line, error bars = SEM). Harmaline was injected at *t* = 0. **(D)** *Maximum tremor index for each behavioral session.* Maximum tremor response (mean value for *n* = 5 animals, error bars = SEM) decreased across sessions. ^∗^indicates statistical significance (*p* < 0.05, paired-*t* test).

Interleaved with the fMRI sessions, we measured harmaline-induced tremor on three separate occasions. Harmaline immediately generated a repetitive motion response (Figure 5A). Fast Fourier transformation of raw accelerometer data reveals a frequency range of the repetitive motion response between 10 and 16 Hz with a prominent peak near 13 Hz (red line), which was classified as tremor (Figure 5B; Lee et al., 2018). A time course of harmaline-induced tremor with repetitive drug injections was also analyzed (Figure 5C). Tremor was initiated immediately after drug injection and maintained over an hour. As previously reported, the repeated administration of harmaline was associated with a progressive reduction in drug-induced tremor (Lutes et al., 1988; Wang and Fowler, 2001). Both maximum tremor index (*p* = 0.046 for 2nd vs. 4th sessions and *p* = 0.048 for 2nd vs. 6th sessions, paired *t*-test) and tremor duration declined with successive drug injections (*p* < 0.0005, one-way ANOVA).

**FIGURE 6:**
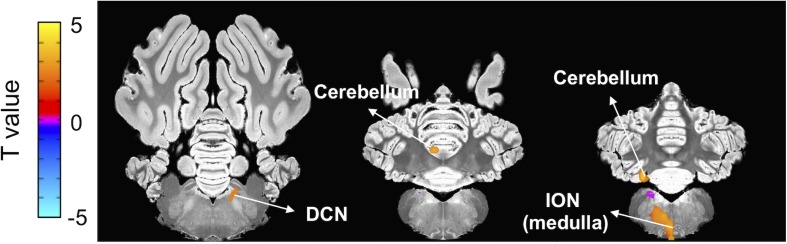
Correlation between fMRI beta coefficients and tremor index. Voxel-wise regression analysis using beta coefficients and tremor indices revealed a significant positive correlation between ION, cerebellum and DCN activity and tremor index (*p* < 0.05, uncorrected) (Table 3).

Next, to assess the correlation between changes in the brain activity observed during the three fMRI sessions and the tremor severity, voxel-wise regression analysis was performed. Beta coefficients were determined from the fMRI data and compared to the maximum value of tremor index for each animal during the interleaved behavioral sessions. As shown in Figure 6 and Table 3, there was a significant positive correlation of BOLD signal in the ION, cerebellum, and DCN regions with tremor index, whereby greater activation in these regions was associated with increased tremor severity (*p* < 0.05).

**TABLE 3:** Regions of significant correlation between harmaline induced fMRI activation and tremor score.

**Hemisphere**	**Area**	***x***	***y***	***z***	**Cluster size**	***t***
L	Olivary nuclei; medulla	0	21.1	–13.9	21	3.029
L	Cerebellum	7	21.1	–4.1	13	3.36
R	Deep cerebellar nucleus	−4.2	11.3	–8.2	6	2.27
L	Cerebellum	2.8	19.7	1.5	6	3.11

*L, left; R, right.*

### Functional Correlation Changes in Regions of Interest Level

**FIGURE 7:**
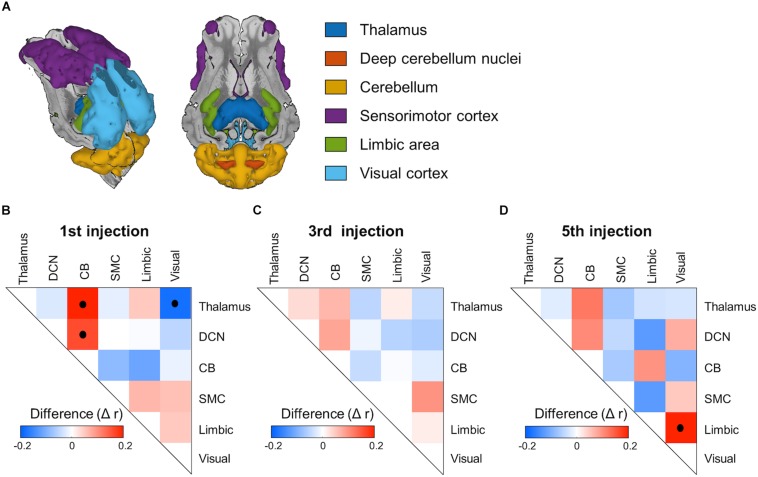
ROI-based functional network connectivity changes induced by harmaline injection. **(A)** *Regions of interest (ROI).* Color representations of ROI, including thalamus, deep cerebellum nuclei, cerebellum, sensorimotor cortex, limbic area and visual cortex. **(B–D)** Adjacency matrices of inter-ROI correlation for each fMRI session. The inter-ROI correlation was derived as partial correlation coefficient between mean voxel signals within each ROI. Post-saline injection matrices were subtracted from post-harmaline injection matrices to isolate functional connectivity changes specific to harmaline injection. Black dots inside the matrixes indicate statistical significance (*p* < 0.001, permutation test, *n* = 2000).

To illustrate the harmaline-induced changes in functional correlation between ROIs, adjacency matrices of ROI-level functional correlations were computed (Figure 7). With the first injection of harmaline, the correlations between activity in cerebellum-thalamus and between cerebellum-DCN were significantly increased (*p* < 0.001; Figure 7B). However, the magnitude of these correlations decreased across sessions. The correlation between cerebellar and thalamic activity was 0.20, 0.06, and 0.12, and the correlation between cerebellar and DCN activity was 0.16, 0.08 and 0.10 for first, third and fifth injections, respectively. Conversely, the correlation between visual cortex and thalamic activity was significantly negative (*p* < 0.001) and the magnitude of this negative correlation also decreased across sessions. Finally, a significant correlation between activity in the visual cortex and limbic regions was noted only with the fifth injection (*p* < 0.001).

## Discussion

Because ET is one of the most common and debilitating neurodegenerative disorders, functional mapping and identification of tremorogenic mechanisms have emerged as important clinical and scientific questions. In this study, we used ph-fMRI to evaluate at a system level the modulation of brain activity induced by harmaline administration in a large animal model. We observed significant BOLD signal changes at several key nodes within the olivo-cerebello-thalamo-cortical motor control circuitry, including ION, cerebellum, thalamus, caudate, and premotor cortex immediately following harmaline injection. In addition, harmaline administration was associated with increased BOLD signal in the caudate and visual cortex. These results are consistent with hypothesized mechanisms of ET and help to validate the harmaline tremor model in swine as an important preclinical model to advance understanding of tremor generation mechanisms and trial new therapeutic approaches.

Harmaline has long been used to develop animal models of tremor in multiple different species including mice, rats, pigs, and non-human primates (Goldstein et al., 1969; Miwa, 2007; Lee et al., 2018). The characteristics of harmaline-induced tremor, including its predominantly action-based components and its improvement with propranolol and ethanol, are similar to those of ET in humans. In addition, β-carboline derivatives are observed to increase in the blood and brain of human patients with ET (Boecker et al., 1996). However, the degree to which the pathogenesis of harmaline-induced tremor recapitulates clinical ET has remained controversial. The studies performed here establish a critical link between animal investigation and the clinical literature.

### Cerebellar Activation

In both physiology studies in rodent models and fMRI studies in humans with ET, the thalamo-cortical-cerebellar motor network has been implicated in tremor genesis (Bucher et al., 1997; Buijink et al., 2015b; Gallea et al., 2015). Functional imaging studies in patients most consistently suggest a major role of cerebellum in the pathophysiology of tremor (Boecker and Brooks, 1998; Schnitzler et al., 2009). Furthermore, cellular analysis of the cerebellum of ET patients also reveals pathological abnormalities (Louis et al., 2011a, b). Consistent with these findings in patients with ET, our fMRI study captured the harmaline-induced activation of the cerebellum, especially DCN (Figure 3). This supports the possibility that harmaline-induced tremor in the pig may share underlying mechanisms of ET pathogenesis, particularly involvement of cerebellar pathways.

### Thalamic Activation

As outlined in the introduction, there is controversy surrounding the relative contribution of thalamus to ET versus harmaline-induced tremor. Previous animal studies have shown an independence of harmaline-induced tremor from thalamic and cortical involvement (Battista et al., 1970; Villablanca and Riobó, 1970; Lamarre et al., 1975). Importantly, the present study reveals similar involvement along all nodes of the cerebello-thalamo-cortical network implicated in human ET, including thalamus. However, the activated area in the thalamus was not identical to the thalamic area believed to be involved in humans with ET. For neurosurgical treatment of ET, DBS or lesioning of the ventral intermediate nucleus (VIM) of the thalamus have been used. This is based on studies that have demonstrated a relationship between VIM thalamus and tremor oscillatory networks (Hassler and Riechert, 1954) as well as connectivity alterations between the VIM thalamus and other brain regions within the thalamo-cortical-cerebellar network in patients with ET (Fang et al., 2016). However, the therapeutic outcome of thalamic DBS is not always optimal. In the present study, harmaline did significantly activate thalamic regions but not precisely the ventrolateral thalamus, a homolog area of the human VIM thalamus (Figure 3). While this finding is not entirely consistent with our prediction that thalamic activation would be localized in the ventrolateral thalamus, the difference in precise region of thalamic involvement may align with existing controversy in the literature over how best to target thalamic stimulation in order to optimize patient outcome (Akram et al., 2018; Gunalan et al., 2018) These studies highlight individual connectivity as an important factor in targeting of therapy. Therefore, although thalamic regions, particularly regions near the VIM, appear to play a critical role in tremor the exact location of thalamic involvement in individual patients may be difficult to determine based solely on anatomic regional delineations without consideration of connectivity. The issue of thalamic involvement in ET and precise targeting of thalamic regions for therapeutic intervention remain a subject of ongoing investigation, making it difficult to draw definitive conclusions at this time based solely on our finding of thalamic activation. To further refine treatment, it will be important to identify the precise thalamic target for DBS and other neuromodulation treatment options including thalamotomy and high-intensity focused ultrasound. An advantage of the swine model is that it enables the use of imaging and neuromodulation techniques that are easily translatable to human patients (Min et al., 2012; Paek et al., 2015) and is thus ideally suited for such investigations.

### Inferior Olivary Nucleus (ION) Activation

We also observed activation of ION with harmaline administration (Figure 3). Prior to the emergence of fMRI as a popular method to visualize brain activity, identification of mechanisms of tremor genesis was performed using electrophysiology and immunohistochemistry during harmaline injection in small animal models of harmaline-induced tremor. Those studies suggest that modulation of Purkinje cell firing by ION oscillatory activity in turn changes cerebellar to cortical transmission driving tremor (Llinás and Volkind, 1973; Lorden et al., 1988; Miwa et al., 2000; Park et al., 2010), thus implicating the ION as a main effector of tremor genesis. However, in clinical human fMRI experiments, ION activation has not been readily detected. In fact, only one study has described BOLD activation changes in the olivary nucleus related to ET (Buijink et al., 2015a). This study found BOLD activation in the medulla adjacent to but not precisely within the ION. However, the ION is relatively small area and due to volume averaging effects can be difficult to be detect through fMRI; therefore absence of detectable BOLD activation in this region is not sufficient evidence of its lack of significance. Given that we were also able to demonstrate ION activation using fMRI in a large animal model (Figure 3), which is anatomically more similar to humans than a rodent model, the functional involvement of the ION in ET warrants further investigation.

### Non-motor Activation

In addition to the motor symptoms that are the primary manifestations of ET, non-motor neuropsychological dysfunction has been reported in ET. For example, an fMRI study to assess attentional control and evaluate executive function found that patients with ET showed a greater magnitude of brain response in the dorsolateral prefrontal cortex and in the inferior parietal cortex as compared to controls, even though patient performance was similar to the control group (Cerasa et al., 2010). This suggests that patients with ET require additional cognitive effort to achieve comparable performance levels on tests of attentional control. In the present study, during the first injection session, there was significant activation of non-motor circuitry including hippocampus, caudate nucleus and amygdala (Figures 3, 4). The harmaline-associated BOLD activation in this non-motor circuitry may explain associated neuropsychological and/or affective symptoms, and this would be consistent with reports that a harmaline can have effects on catecholamines (Moloudizargari et al., 2013). However, these non-motor findings were incidental in the present study and the relationship between these imaging results and behavioral non-motor dysfunction in human ET warrants further systemic investigation.

### Tolerance to Harmaline and Implications of the Swine Model

Finally, use of harmaline in the large animal model has been limited by tolerance effects to repeated harmaline injections. In the present study, consistent with previous reports (Lutes et al., 1988; Wang and Fowler, 2001), harmaline-induced tremor was reduced with repeated injections of harmaline (Figure 5), and was correlated with a similar reduction in BOLD activation of ION, cerebellum, and DCN (Figures 4, 6). Moreover, the correlations in functional activity between cerebellum and thalamus and between cerebellum and DCN decreased as the injection was repeated. This result would be expected given previous findings of harmaline-induced neurodegeneration of Purkinje cells in cerebellum and ION (O’hearn and Molliver, 1993; O’Hearn and Molliver, 1997; Miwa, 2007). It is thought that the excitatory projections (i.e., climbing fibers) originating from the ION may release excessive glutamate, resulting to a loss of Purkinje cells, thus explaining the development of tolerance. Therefore, it is likely that ION and cerebellum cell loss due to repeated harmaline injection are responsible for the observed decrement of BOLD activity in those regions. This highlights the importance of limiting the number of injections an animal receives and/or counterbalancing the experimental design when this model is used for preclinical studies of tremor therapies.

Yet this effect imposes important constraints on the use of large animals for studies of ET using harmaline. Because of the increased economic and ethical burden associated with many large animal models such as primates, underlying mechanisms of harmaline-induced tremogenesis in these models have not been thoroughly investigated. However, the larger brain size conversely makes these models ideal candidates for mapping of global brain activity via functional imaging (Fang et al., 2006; Min et al., 2012; Paek et al., 2015) and for developing new therapeutic techniques that are rapidly translatable to patients with ET. The swine model represents an optimal balance between the advantages of larger brain size and greater homology to the human brain than rodent models and disadvantages of added financial and ethical burden, allowing for larger sample sizes and more extensive preclinical investigations.

### Limitations

It is important, however, to consider some limitations and technical points constraining interpretation of these findings. First, the behavioral analysis of tremor and the fMRI monitoring were conducted in separate sessions due to the need for anesthesia use to facilitate fMRI acquisition, thus limiting a direct correlation between the two measurements. However, the interleaved pattern of the sessions and the within subjects design allowed for some correlation of these two datasets.

Second, a significant obstacle faced during the investigation of functional anatomy associated with harmaline-induced tremor was the induction of significant BOLD signal fluctuations (Figure 2) due to the systemic physiologic effects of intravenously administered harmaline (Aarons et al., 1977; Berrougui et al., 2006). In human fMRI studies it is customary to reduce non-neuronal signal changes by global signal regression based on tissue-based signals (ventricles, white and gray matter) (Power et al., 2014). However, given the low spatial resolution of the EPI data in combination with the smaller size of the pig brain as compared the human brain, few white matter and ventricular voxels survived during erosion after tissue segmentation. Hence, to correct for these systemic physiologic influences on BOLD activity we continuously measured heart and respiration rate and utilized these in the post-imaging process to attenuate the changes in BOLD signal that could be attributed to cardiovascular changes, thus unmasking the harmaline-induced functional connectivity changes. Another confounder was the possibility of BOLD artifacts related to expansion in blood volume as a consequence of bolus harmaline injection. However, a saline injection of equivalent volume did not induce significant changes in BOLD activity.

An additional difference between this study and the human literature involves the use of anesthesia. Because functional neuroimaging is difficult to apply in conscious animals, anesthesia is essential. In this study, telazol and xylazine were used for initial induction and isoflurane was used to maintain the general anesthesia. It is important to note that awake fMRI may yield different results from those in the anesthetized state (Sicard et al., 2003; Li et al., 2013). However, the dose of anesthetic was minimized to reduce effects on the neural activity, and previous studies have likewise shown robust external stimulation-dependent BOLD responses and electrophysiological changes in the anesthetized state (Masamoto et al., 2006; Angenstein et al., 2010; Min et al., 2012; Lee et al., 2015).

A final potential limitation of intravenous injection is that harmaline easily penetrates the blood-brain barrier. Although this certainly enables harmaline to function as a tremor induction agent, it could lead to non-specific global BOLD signal changes unrelated to tremor-associated changes in neural activity. Yet, our fMRI data shows that harmaline effect was highly limited to specific brain areas including the ION, deep cerebellum and thalamus rather than non-selective global activation. Furthermore, the reduction in behavioral measurement of tremor with repeated injection correlated with reduction in BOLD activation of those same brain regions. Harmaline has been demonstrated to modulate the oscillatory activity of the Cav3.1 T-type Ca^2+^ channel expressed by ION neurons and has also been shown to be mediated through the gap junction, connexin-36 (Long et al., 2002). If this is true, one might expect that all brain areas where the specific calcium channels and/or gaps junction are expressed to be directly affected by harmaline and potentially demonstrate a BOLD signal response. However, those areas include amygdala, cerebral cortex and rostral hypothalamus (Talley et al., 1999), lateral reticular nucleus, and hippocampus (Parenti et al., 2000), which were not all strongly activated by harmaline in our study. How harmaline selectively affect the neurons in ION compared to other areas which express T-type Ca^2+^ channel and/or Cx36 needs to be further investigated.

### Summary

Overall, these results demonstrate a significant activation of the olivo-cerebello-thalamo-cortical network associated with harmaline-induced tremor in a large animal model, implicating this network as the primary mediator of the pathology as well as a potential therapeutic target. Furthermore, the results described here reveal a strong overlap between the patterns of changes in functional connectivity in the harmaline-induced tremor model in the pig and that described in studies of patients with ET. This further validates harmaline as a plausible model for continued study of global brain mechanisms involved in ET and its treatment. Most critically, the innovative combination of a relatively inexpensive large animal model with pharmacologic fMRI opens up multiple avenues for further study.

## Data Availability

The raw data supporting the conclusions of this manuscript will be made available by the authors, without undue reservation, to any qualified researcher.

## Author Contributions

JeL, IK, JiL, and S-YC performed the animal training and behavior data collection. JiL, HJ, H-KM, and M-HI acquired the imaging data and optimized the imaging protocol. JeL and HJ analyzed the data. S-YC, and HJ conceived and designed the experiments. JeL, EK, HJ, and S-YC prepared the manuscript.

## Conflict of Interest Statement

The authors declare that the research was conducted in the absence of any commercial or financial relationships that could be construed as a potential conflict of interest.

## Supplementary Material

FIGURE S1 **(A,C,E)** BOLD signal change in 1st, 3rd and 5th injection, respectively. Harmaline injection was initiated approximately 4 min for 40 s (shaded area represents the injection period). **(B,D,F)** BOLD signal traces after de-noise in 1st, 3rd and 5th injection, respectively.

FIGURE S2 Overall harmaline-induced activation map across whole sessions based on different block length (One-sample *t*-test, *q* < 0.0005, FDR corrected).
