# Supplementary figures and images for: Mapping BOLD Activation by Pharmacologically Evoked Tremor in Swine
Source: Front Neurosci. 2019 Sep 18;13:985. doi: 10.3389/fnins.2019.00985 (PMC6759958; doi:10.3389/fnins.2019.00985)

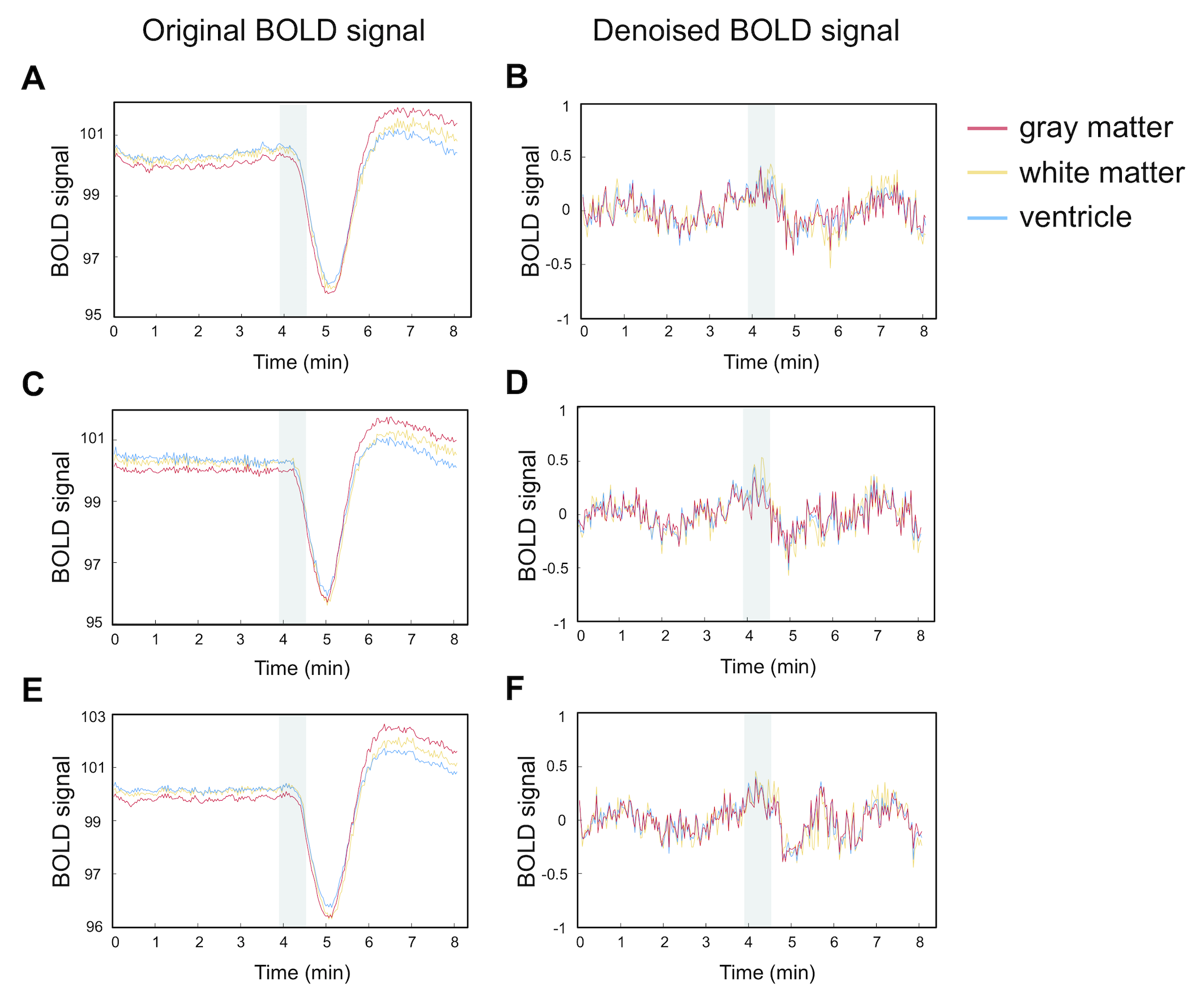

Supplement: FIGURE S1 — (A,C,E) BOLD signal change in 1st, 3rd and 5th injection, respectively. Harmaline injection was initiated approximately 4 min for 40 s (shaded area represents the injection period). (B,D,F) BOLD signal traces after de-noise in 1st, 3rd and 5th injection, respectively. [file Image_1.TIFF]

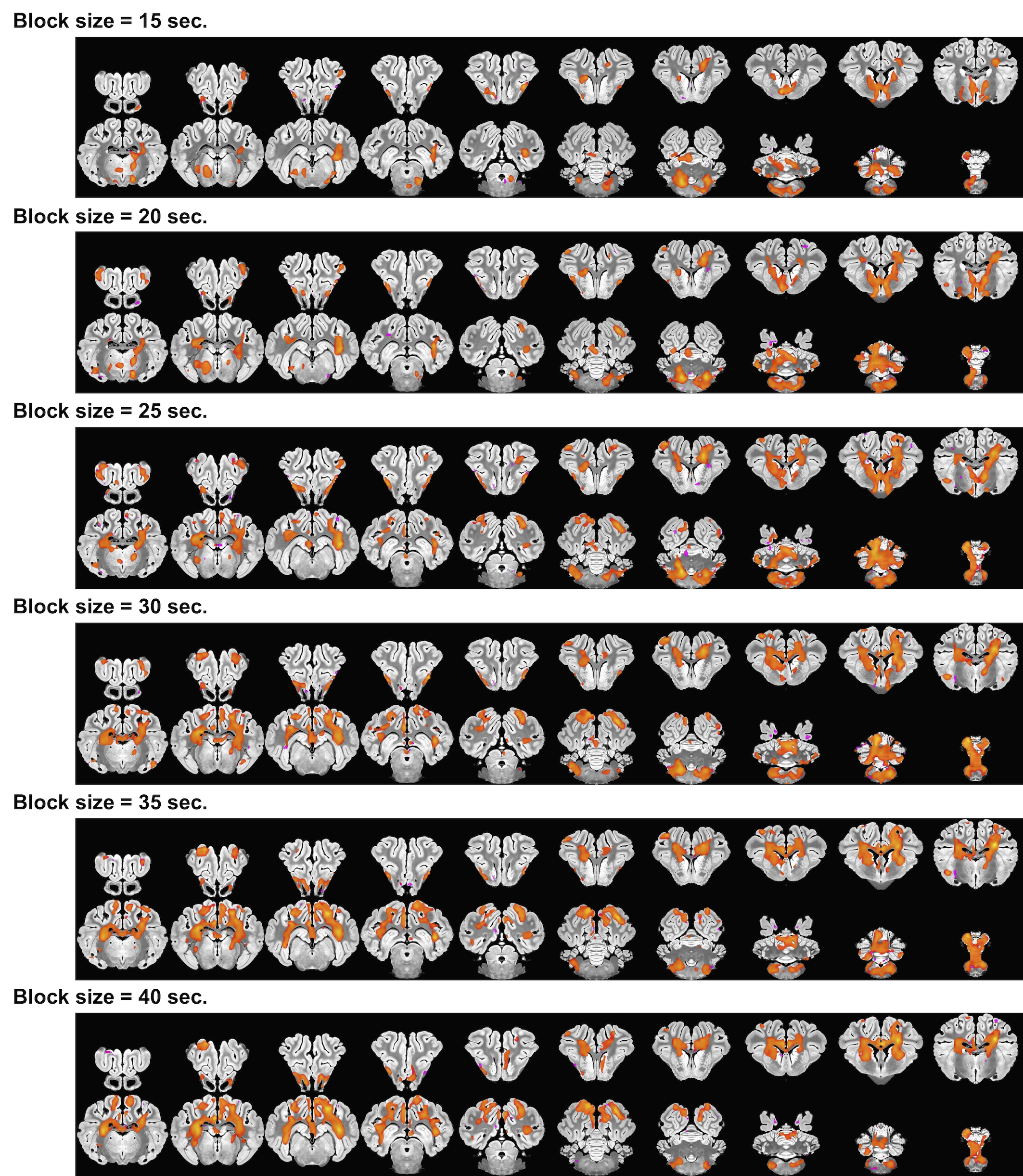

Supplement: FIGURE S2 — Overall harmaline-induced activation map across whole sessions based on different block length (One-sample t-test, q < 0.0005, FDR corrected). [file Image_2.TIFF]
